# Hepatoprotective Effects of *Aureobasidium pullulans* Derived β 1,3–1,6 Glucans in a Murine Model of Non-alcoholic Steatohepatitis

**DOI:** 10.1016/j.jceh.2022.06.008

**Published:** 2022-06-27

**Authors:** Nobunao Ikewaki, Gary A. Levy, Gene Kurosawa, Masaru Iwasaki, Vidyasagar D. Dedeepiya, Suryaprakash Vaddi, Rajappa Senthilkumar, Senthilkumar Preethy, Samuel J.K. Abraham

**Affiliations:** ∗ Department of Medical Life Science, Kyushu University of Health and Welfare, Japan; † Institute of Immunology, Junsei Educational Institute, Nobeoka, Miyazaki, Japan; ‡ Medicine and Immunology, University of Toronto, Ontario, Canada; § Department of Academic Research Support Promotion Facility, Center for Research Promotion and Support, Fujita Health University, Aichi, Japan; ‖ MabGenesis KK, Nagoya, Japan; ¶ Centre for Advancing Clinical Research (CACR), University of Yamanashi - School of Medicine, Chuo, Japan; # Mary-Yoshio Translational Hexagon (MYTH), Nichi-In Centre for Regenerative Medicine (NCRM), Chennai, India; ∗∗ Department of Urology, Yashoda Hospitals, Hyderabad, India; †† Fujio-Eiji Academic Terrain (FEAT), Nichi-In Centre for Regenerative Medicine (NCRM), Chennai, India; ‡‡ Antony- Xavier Interdisciplinary Scholastics (AXIS), GN Corporation Co. Ltd., Kofu, Japan

**Keywords:** non-alcoholic fatty liver disease (NAFLD), non-alcoholic steatohepatitis (NASH), beta-glucans, anti-fibrotic, anti-inflammatory, hepatoprotective, telmisartan, ALT, Alanine aminotransferase, ARRIVE, Animal Research: Reporting of In Vivo Experiments, IL, Interleukin, MCP-1, Monocyte chemoattractant protein-1, PPAR, Peroxisome proliferator-activated receptor, NAFLD, Non-alcoholic fatty liver disease, NAS, NAFLD Activity Score, NASH, Non-alcoholic steatohepatitis, αSMA, Smooth muscle alpha-actin, STAM, Stelic Animal Model, TGF-β, Transforming growth factor beta, TIMPs, Tissue inhibitors of matrix metalloproteinases, TNF-α, Tumor necrosis factor alpha

## Abstract

**Background:**

Non-alcoholic fatty liver disease (NAFLD) and non-alcoholic steatohepatitis (NASH) are highly prevalent conditions characterized by inflammation and fibrosis of the liver, which can progress to cirrhosis and hepatocellular carcinoma if left untreated. Conventional modalities are mainly symptomatic, with no definite solution. Beta-glucan-based biological response modifiers are a potential strategy in lieu of their beneficial metabolic effects. *Aureobasidium pullulans* strains AFO-202 and N-163 beta-glucans were evaluated for anti-fibrotic and anti-inflammatory hepatoprotective potentials in a NASH animal model in this study.

**Methods:**

In the STAM™ murine model of NASH, five groups were studied for 8 weeks: (1) vehicle (RO water), (2) AFO-202 beta-glucan; (3) N-163 beta-glucan, (4) AFO-202+N-163 beta-glucan, and (5) telmisartan (standard pharmacological intervention). Evaluation of biochemical parameters in plasma and hepatic histology including Sirius red staining and F4/80 immunostaining were performed.

**Results:**

AFO-202 beta-glucan significantly decreased inflammation-associated hepatic cell ballooning and steatosis. N-163 beta-glucan decreased fibrosis and inflammation significantly (*P* value < 0.05). The combination of AFO-202 with N-163 significantly decreased the NAFLD Activity Score (NAS) compared with other groups.

**Conclusion:**

This preclinical study supports the potential of N-163 and AFO-202 beta-glucans alone or in combination as potential preventive and therapeutic agent(s), for NASH.

## Graphical abstract

**Figure:**
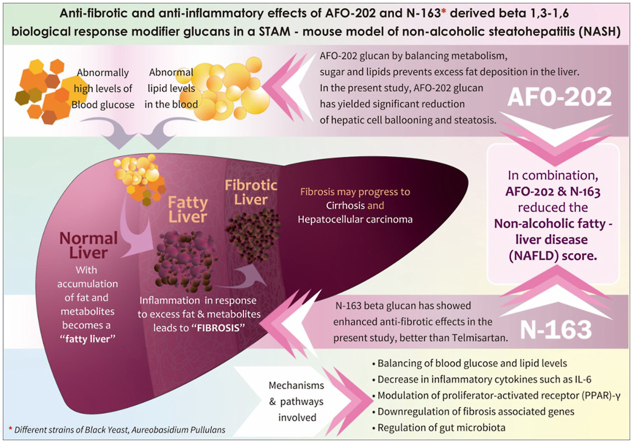

Non-alcoholic fatty liver disease (NAFLD) refers to a group of conditions in which there is excess fat accumulation on the liver in people who drink little or no alcohol.^1^ Non-alcoholic steatohepatitis (NASH) is a severe form of NAFLD. NAFLD or NASH progresses to liver fibrosis, liver cirrhosis, liver failure, or carcinoma if not treated. Increased prevalence of obesity and metabolic syndrome, diabetes, and dysregulated lipid levels, all add to the problem of NAFLD and NASH. NAFLD and NASH involve pathologic features such as hepatic steatosis, lobular inflammation, hepatocellular ballooning, and liver fibrosis, which ultimately lead to cirrhosis.^1^^,^^2^ There are no definite treatments for NASH. Conventional approaches aim to address the underlying condition such as diabetes and metabolic disease with lifestyle changes, weight reduction, specific medication such as thiazolidinediones, lipid-lowering agents, cytoprotective agents, and antioxidants such as vitamin E.^3^ Angiotensin receptor blockers such as telmisartan, which act by modulating transcription factor peroxisome proliferator-activated receptor (PPAR)-γ activity,^4^ thereby increasing insulin sensitivity, are increasingly being advocated. However, the underlying etiology and disease pathogenesis need more holistic approaches.

Animal models have proven to be highly useful to investigate the etiopathogenesis of a number of human diseases.^5^ Stelic Animal Model (STAM™) is an animal model that recapitulates the disease progression of that which occurs in human NASH/HCC.^6^ In this model, C57BL/6 mice aged 2 days are given a single dose of streptozotocin to reduce the insulin secretory capacity. When the mice turn 4 weeks of age they are started on a high-fat diet (HFD) feeding. This model has a background of late type 2 diabetes, which progresses into fatty liver, NASH, fibrosis and consequently HCC.^2^^,^^6^ Since this model recapitulates the full spectrum of human NAFLD ranging from steatosis to NASH and hepatic fibrosis apart from the histological phenotypes similar to those seen in human clinical samples^7^ allowing the same scoring system (NAFLD activity score; NAS) to be used to assess the severity of the disease, makes this a highly recommended model for NASH studies. In this study, we have employed the STAM – animal model to study the hepatoprotective anti-fibrotic and anti-inflammatory effects of beta-glucans from a black yeast, *Aureobasidium pullulans*. Beta-glucans are potent biological response modifiers that have been proven to be effective in modulating dysregulated metabolism by regulating blood glucose and lipid levels. The *Aureobasidium pullulans* AFO-202 strain-derived 1,3–1,6 beta-glucan has been demonstrated to decrease HbA1c to normal values and decrease fasting and post-prandial blood glucose in human clinical studies.^8^^,^^9^ This GMP-manufactured beta-glucan has been proven to regulate lipid levels of triglycerides, total cholesterol, and HDL cholesterol in another human clinical study.^10^ Another variant of the 1,3–1,6 beta-glucan has been derived from a novel strain, N-163 of *Aureobasidium pullulans*, which in in vitro studies has shown to have a positive effect on lipid metabolism.^11^ In the present study, we report the anti-fibrotic and anti-inflammatory hepatoprotective effects of AFO-202 and N-163-strains derived beta-glucan individually and in combination, in STAM mice.

## Materials and methods

### Mice

The study is reported in accordance with Animal Research: Reporting of In Vivo Experiments (ARRIVE) guidelines. C57BL/6J mice were obtained from Japan SLC, Inc. (Japan). All animals used in this study were cared for under the following guidelines: Act on Welfare and Management of Animals (Ministry of the Environment, Japan, Act No. 105 of October 1, 1973), standards relating to the care and management of laboratory animals and relief of pain (Notice No.88 of the Ministry of the Environment, Japan, April 28, 2006) and the guidelines for proper conduct of animal experiments (Science Council of Japan, June 1, 2006). Protocol approvals were obtained from SMC Laboratories, Japan's IACUC (Study reference no: SP_SLMN128-2107-6_1). Mice were maintained in a specific pathogen-free (SPF) facility under controlled conditions of temperature (23 ± 3 °C), humidity (50 ± 20%), lighting (12-h artificial light and dark cycles; light from 8:00 to 20:00) and air exchange.

The STAM model of NASH was induced as previously described.^6^Mice were given a single subcutaneous injection of 200 μg streptozotocin (STZ, Sigma–Aldrich, USA) solution 2 days after birth and fed with a HFD (57 kcal% fat, Cat# HFD32, CLEA Japan, Inc., Japan) from 4 to 9 weeks of age.^2^^,^^6^ All mice develop liver steatosis and diabetes, and at 3 weeks mice had established steatohepatitis, histologically.^2^^,^^7^

### Study Groups

There were five study groups, described below. Eight mice were included in each study group.

#### Group 1: Vehicle

Eight NASH mice were orally administered the vehicle (RO water) in a volume of 5 mL/kg once daily from 6 to 9 weeks of age.

#### Nichi Glucan Groups

The dose of Nichi glucan was decided based on the earlier studies of AFO-202 strain-derived beta-glucan in human healthy volunteers and subjects with lifestyle disorders (diabetes dyslipidemia)^8^, ^9^, ^10^ and N-163 strain-derived betaglucan in healthy volunteers.^12^

#### Group 2: AFO-202 Beta-Glucan

Eight NASH mice were orally administered the vehicle supplemented with AFO-202 beta-glucan at a dose of 1 mg/kg in a volume of 5 mL/kg once daily from 6 to 9 weeks of age.

#### Group 3: N-163 Beta-Glucan

Eight NASH mice were orally administered the vehicle supplemented with N-163 beta-glucan at a dose of 1 mg/kg in a volume of 5 mL/kg once daily from 6 to 9 weeks of age.

#### Group 4: AFO-202 Beta-Glucan + N-163 Beta-Glucan

Eight NASH mice were orally administered the vehicle supplemented with AFO-202 beta-glucan at a dose of 1 mg/kg in a volume of 5 mL/kg once daily and orally administered the vehicle supplemented with N-163 beta-glucan at a dose of 1 mg/kg in a volume of 5 mL/kg once daily from 6 to 9 weeks of age.

#### Group 5: Telmisartan

Eight NASH mice were orally administered the vehicle supplemented with telmisartan at a dose of 10 mg/kg once daily from 6 to 9 weeks of age.

Telmisartan, which has been reported to have antisteatotic, anti-inflammatory, and antifibrotic effects in STAM model, was used as the positive comparator.

AFO-202 and N-163 beta-glucan were provided by GN Corporation Co Ltd. Telmisartan (Micardis®) was purchased from Boehringer Ingelheim GmbH (Germany).

### Preparation of Test Substances

#### AFO-202 Beta-Glucan and N-163 Beta-Glucan

AFO-202 beta-glucan or N-163 beta-glucan was mixed in the required amount of RO water and stirred until it completely dissolved. The solution was dispensed into 7 tubes and stored at 4 °C until the day of administration. The dosing formulations were stirred prior to administration. The dosing formulations were used within 7 days.

#### Telmisartan

Formulations were freshly prepared prior to administration. One tablet of telmisartan was transferred into mortar and triturated using a pestle by adding RO water gradually to get 1 mg/mL of homogeneous suspension.

### Randomization

NASH model mice were randomized into five groups of eight mice at 6 weeks of age based on their body weight the day before the start of treatment. The randomization was performed by body weight-stratified random sampling using Microsoft Excel. NASH model mice were stratified by their body weight to get the SD and difference in the mean weights among groups as small as possible.

### Animal Monitoring and Sacrifice

Mice were monitored for clinical signs (lethargy, twitching, labored breathing), behavior, and survival. Body weight was recorded daily. Mice were observed for significant clinical signs of toxicity, moribundity, and mortality before and after administration.

The animals were sacrificed at 9 weeks of age by exsanguination through direct cardiac puncture under isoflurane anesthesia (Pfizer Inc.).

At the time of sacrifice, the mice are expected to have reached the steatohepatitis phase of the disease and a mild hepatic fibrotic stage.^2^

If an animal showed >25% body weight loss within a week or >20% body weight loss compared with the previous day, the animal was euthanized ahead of study termination, and samples were not collected. If it showed a moribundity sign, such as prone position, the animal was euthanized ahead of study termination, and samples were not collected.

### Sample collection

The following samples were collected and stored.

• Frozen plasma
• SNAP frozen liver
• Paraffin-embedded liver
• OCT-embedded liver

#### Preparation of Plasma Samples

At study termination, non-fasting blood was collected through direct cardiac puncture using pre-cooled syringes. The collected blood was transferred in pre-cooled polypropylene tubes with anticoagulant (Novo-Heparin) and stored on ice until centrifugation. The blood samples were centrifuged at 1000×*g* for 15 min at 4 °C. The supernatant was collected and stored at −80 °C for biochemistry and evaluation.

#### Preparation of Liver Samples

After sacrifice, the whole liver was collected and washed with cold saline. Photos of individual whole livers (parietal side and visceral side) were taken. Liver weight was measured, and liver-to-body weight ratio was calculated. The left lateral lobes of the livers were separated, dissected and stored.

A Liver specimens were stored at −80 °C embedded in optimal cutting temperature (OCT, Sakura Finetek Japan, Japan) compound for immunohistochemistry.
B Liver specimens were fixed in Bouin's solution (Sigma–Aldrich Japan, Japan) for 24 h. After fixation, these specimens were proceeded to paraffin embedding for HE and Sirius red staining.
C Liver specimens were snap frozen in liquid nitrogen and stored at −80 °C for further analysis.

The left and right medial lobes were snap frozen in liquid nitrogen and stored at −80 °C for evaluation.

The right lobe was snap frozen in liquid nitrogen and stored at −80 °C for biochemistry analysis.

The caudate lobe was snap frozen in liquid nitrogen and stored at −80 °C for evaluation.

### Measurement of Plasma Biochemistry

Plasma ALT levels were measured by FUJI DRI-CHEM 7000 (Fujifilm Corporation).

### Measurement of Liver Biochemistry

#### Measurement of Liver Lipid Content

Liver total lipid extracts were obtained by Folch's method.^14^ Liver samples were homogenized in chloroform–methanol (2:1, v/v) and incubated overnight at room temperature. After washing with chloroform–methanol–water (8:4:3, v/v/v), the extracts were evaporated to dryness and dissolved in isopropanol. Liver triglyceride content was measured by the triglyceride E-test (Wako Pure Chemical Industries, Ltd., Japan). Liver free fatty acid content was measured by the NEFA C-test (FUJIFILM Wako Pure Chemical Corporation).

### Histological Analysis

Sections (4 μm) were cut from paraffin blocks of liver tissue using a rotary microtome (Leica Microsystems). After sectioning, each slide was coded with a number for blinded evaluation by the pathologist. Each number was generated using the RAND function of MS Excel, sorted in ascending order and assigned to slides.

#### Histological Analyses

For hematoxylin and eosin (HE) staining, sections were cut from paraffin blocks of liver tissue prefixed in Bouin's solution and stained with Lillie–Mayer's hematoxylin (Muto Pure Chemicals Co., Ltd., Japan) and eosin solution (Wako Pure Chemical Industries).

The NAFLD Activity Score (NAS) was calculated according to the criteria of Kleiner,^13^ as shown in Table 1. For NAS, bright field images of HE-stained sections were captured using a digital camera (DFC295; Leica, Germany) at 50- and 200-fold magnifications. Steatosis score in 1 section/mouse (representative 1 field at 50-fold magnification), inflammation score in 1 section/mouse (representative 1 field around the central vein at 200-fold magnification), and ballooning score in 1 section/mouse (representative 1 field around the central vein at 200-fold magnification) were estimated.

**Table 1:** Definition of NAFLD Activity Score (NAS) Components.

Item	Extent	NAS
Steatosis	Steatosis at 50-fold magnification	
	<5%	0
	5–33%	1
	>33–66%	2
	>66%	3
Lobular inflammation	Estimation of inflammatory foci	
	No foci	0
	<2 foci/200x	1
	2-4 foci/200x	2
	>4 foci/200x	3
Ballooning	Estimation of number of ballooning cells	
	None	0
	Few ballooning cells	1
	Many cells/prominent ballooning	2

NAFLD, Non-alcoholic fatty liver disease; NAS, NAFLD Activity Score.

To visualize collagen deposition, Bouin's fixed liver sections were stained using picro-Sirius red solution (Waldeck, Germany). Briefly, sections were deparaffinized and hydrophilized with xylene, 100-70% alcohol series and RO water, and then treated with 0.03% picro-Sirius red solution (Cat No.: 1A-280) for 60 min. After washing with 0.5% acetic acid solution and RO water, stained sections were dehydrated and cleared with 70–100% alcohol series and xylene, then sealed with Entellan® new (Merck, Germany) and used for observation.

For immunohistochemistry, sections were cut from frozen liver tissues embedded in Tissue-Tek OCT compound and fixed in acetone. Endogenous peroxidase activity was blocked using 0.03% H_2_O_2_ for 5 min, followed by incubation with Block Ace (Dainippon Sumitomo Pharma Co. Ltd., Japan) for 10 min. The sections were incubated with anti-F4/80 antibody at 4 °C overnight. After incubation with a secondary antibody, enzyme–substrate reactions were performed using 3, 3′-diaminobenzidine/H_2_O_2_ solution (Nichirei Bioscience Inc., Japan). The primary antibody used was monoclonal antibody to mouse macrophages (BMA Biomedicals) at a dilution of 100-folds. The peroxidase-based detection system, VECTASTAIN ABC KIT (Vector Laboratories) was used as the secondary antibody staining system.

For quantitative analysis of the fibrosis area and inflammation area, bright field images of Sirius red-stained and F4/80-immunostained sections were captured around the central vein using a digital camera (DFC295; Leica, Germany) at 200-fold magnification, and the positive areas in 5 fields/section were measured using ImageJ software (National Institute of Health, USA).

Quantitative RT-PCR for IL-6 expression was performed on the total RNA extracted from the liver and ileum samples using PCR DICE and TB Green Premix EX Taq II (Takara Bio). The relative mRNA expression level was normalized to that of reference gene 364B (gene symbol: Rplp0).

### Statistical Analysis

Statistical analyses were performed using Prism Software 6 (GraphPad Software, USA). Comparisons were made between the following groups using the Bonferroni multiple comparison test: Group 1 (vehicle) vs. Group 2 (AFO-202 beta-glucan), Group 3 (N-163 beta-glucan), Group 4 (AFO-202 beta-glucan + N-163 beta-glucan), and Group 5 (telmisartan). *P* values < 0.05 were considered statistically significant. Results were expressed as mean ± SD.

A trend or tendency was assumed when a one-sided t-test returned *P* values < 0.1. Comparisons were made between the following groups:

1) Group 1 (vehicle) vs. Group 2 (AFO-202 beta-glucan)
2) Group 1 (vehicle) vs. Group 3 (N-163 beta-glucan)
3) Group 1 (vehicle) vs. Group 4 (AFO-202 beta-glucan + N-163 beta-glucan)
4) Group 1 (vehicle) vs. Group 5 (telmisartan)

## Results

**Figure 1:**
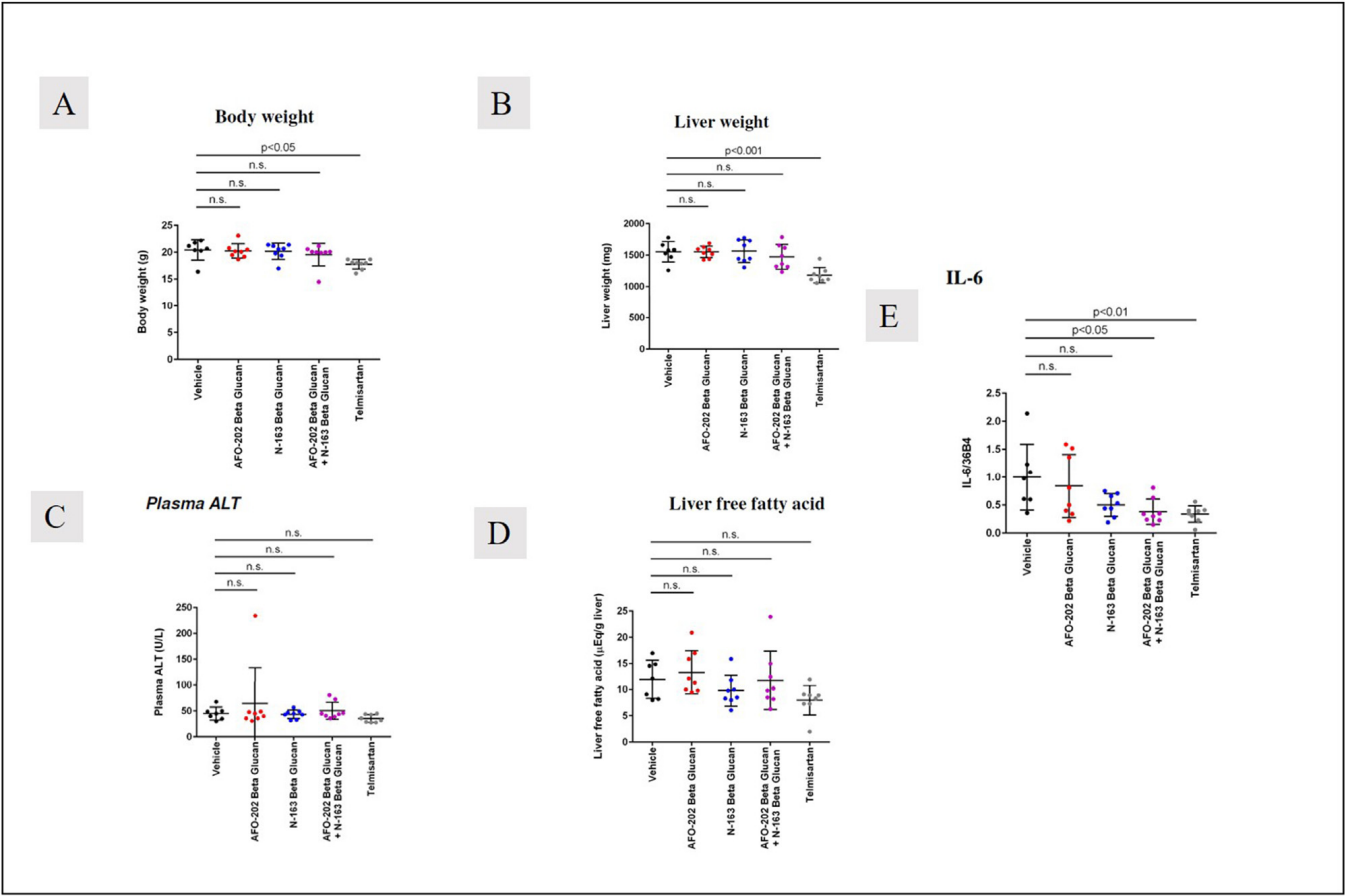
A. Body weight and B. liver weight showing no significant difference between the groups; telmisartan brings down total body weight and liver weight compared to other groups; C. Plasma ALT (mg/dL) and D. Liver fatty acid levels (mEq/g liver) were decreased in the telmisartan and N-163 groups compared to the other groups E. IL-6 mRNA expression levels were significantly decreased in AFO-202+N-163 and telmisartan groups.

There was no significant difference in the body weight and liver weight between the groups (Figure 1 A, B). The mean ± SD of body weight was 20.4 ± 1.9 g in Group 1, 20.3 ± 1.3 g in Group 2, 20.2 ± 1.5 g in Group 3, 19.6 ± 2.1 g in Group 4 and 17.8 ± 0.9 g in Group 5. The mean ± SD liver weight was 1552 ± 162 mg in Group 1, 1552 ± 92 mg in Group 2, 1565 ± 182 mg in Group 3, 1474 ± 197 mg in Group 4 and 1181 ± 123 mg in Group 5.

Plasma ALT levels were lowest in the telmisartan group (Mean ± SD = 36 ± 7 U/L), followed by Group 3 (N-163) (Mean ± SD = 44 ± 8 U/L) (Figure 1C). Similarly, liver free fatty acid levels were lowest in the telmisartan group (Mean ± SD = 8 ± 2.8 (mEq/g liver), followed by Group 3 (N-163) (Mean ± SD = 9.8 ± 3 (mEq/g liver) (Figure 1D). IL-6 mRNA expression levels were significantly decreased in AFO-202+N-163 (Mean ± SD = 0.38 ± 0.22), and Telmisartan groups (Mean ± SD = 0.34 ± 0.15).

**Figure 2:**
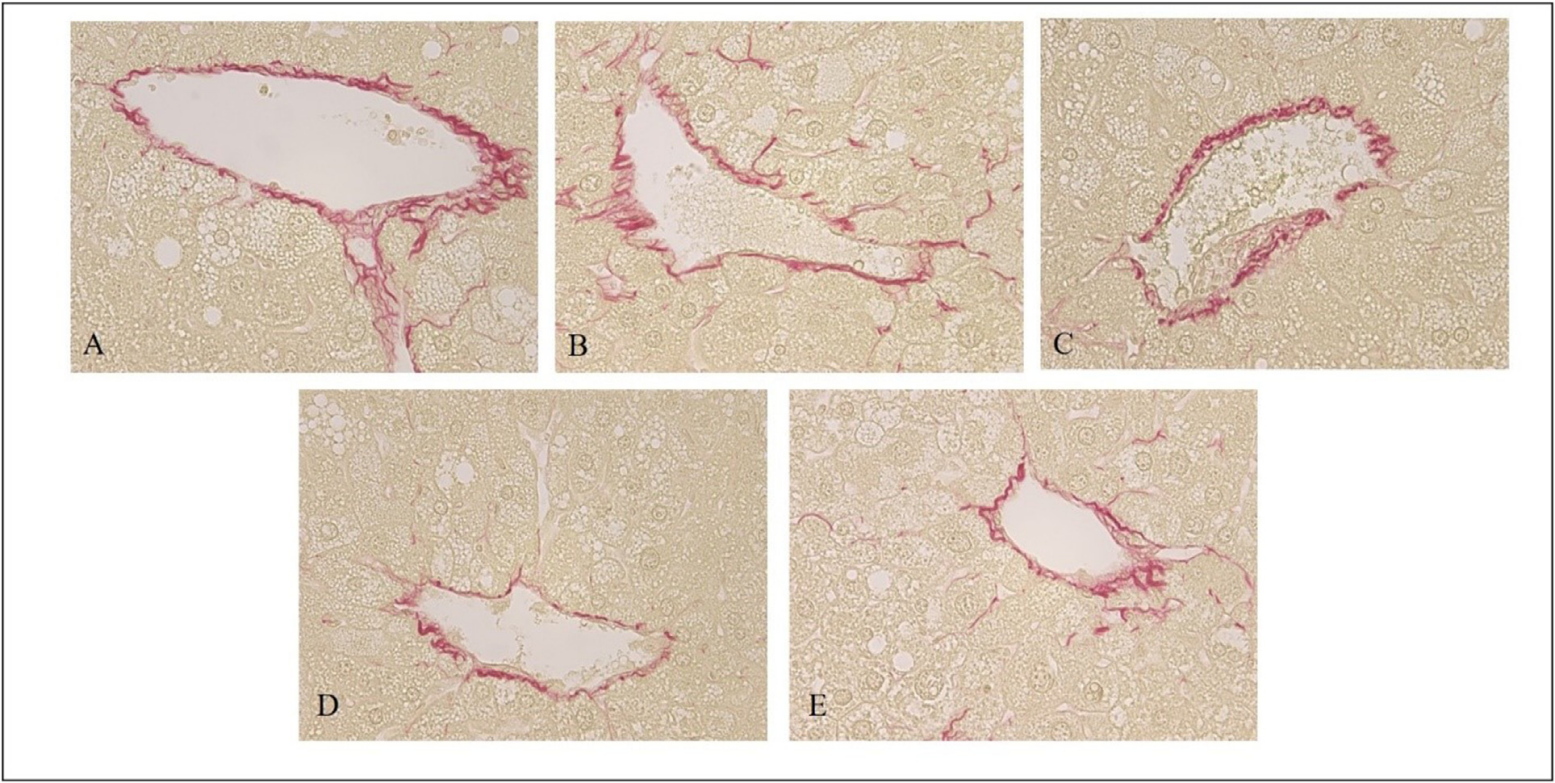
Hepatic fibrosis evaluated with Sirius red staining of A. Vehicle; B. AFO-202; C. N-163; D. AFO 202+N-163 and E. Telmisartan with AFO-202+N-163 (D) and N-163 (C) showing significantly decreased positive staining area compared with all the other groups (magnification: ×400).

**Figure 3:**
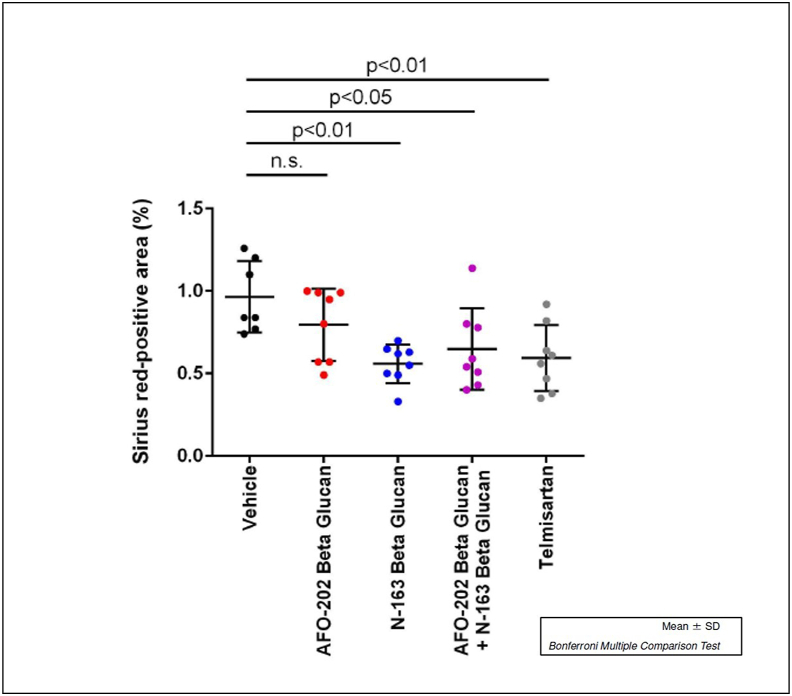
Average positive stained area for fibrosis showing significantly decreased positive staining area in the AFO-202+N-163 and N-163 groups compared with all the other groups.

Representative photomicrographs of Sirius red-stained liver sections are shown in Figure 2. Liver sections from the vehicle group showed increased collagen deposition in the pericentral region of liver lobule. Sirius red-stained images to assess liver damage showed significantly decreased positive staining area in the AFO-202+N-163 and N-163 groups (*P* < 0.05) compared with all the other groups (average positive stained area, AFO-202-0.80 ± 0.22 AFO-202+N-163: 0.65 ± 0.25; N-163: 0.56 ± 0.12; telmisartan: 0.59 ± 0.20; and vehicle: 0.96 ± 0.22) (Figure 2, Figure 3).

**Figure 4:**
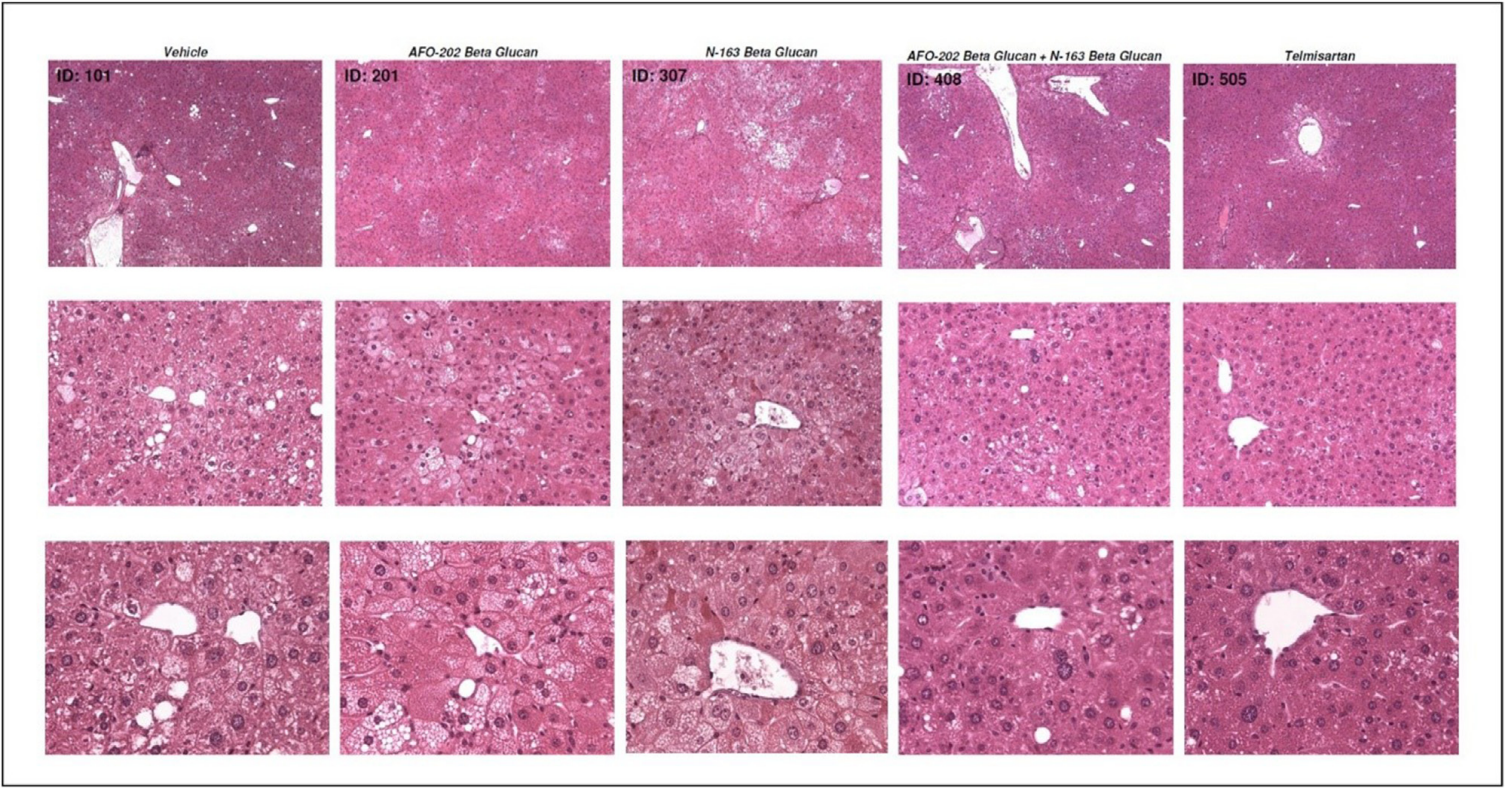
Representative photomicrographs of HE-stained liver sections: Upper panel: magnification = ×50; middle panel: magnification = ×200; lower panel: magnification = ×400.

In H and E staining, liver sections from the vehicle group exhibited micro- and macro-vesicular fat deposition, hepatocellular ballooning and inflammatory cell infiltration. All the beta-glucan treatment groups showed significant decreases in NAS compared with the Vehicle group (Figure 4).

**Figure 5:**
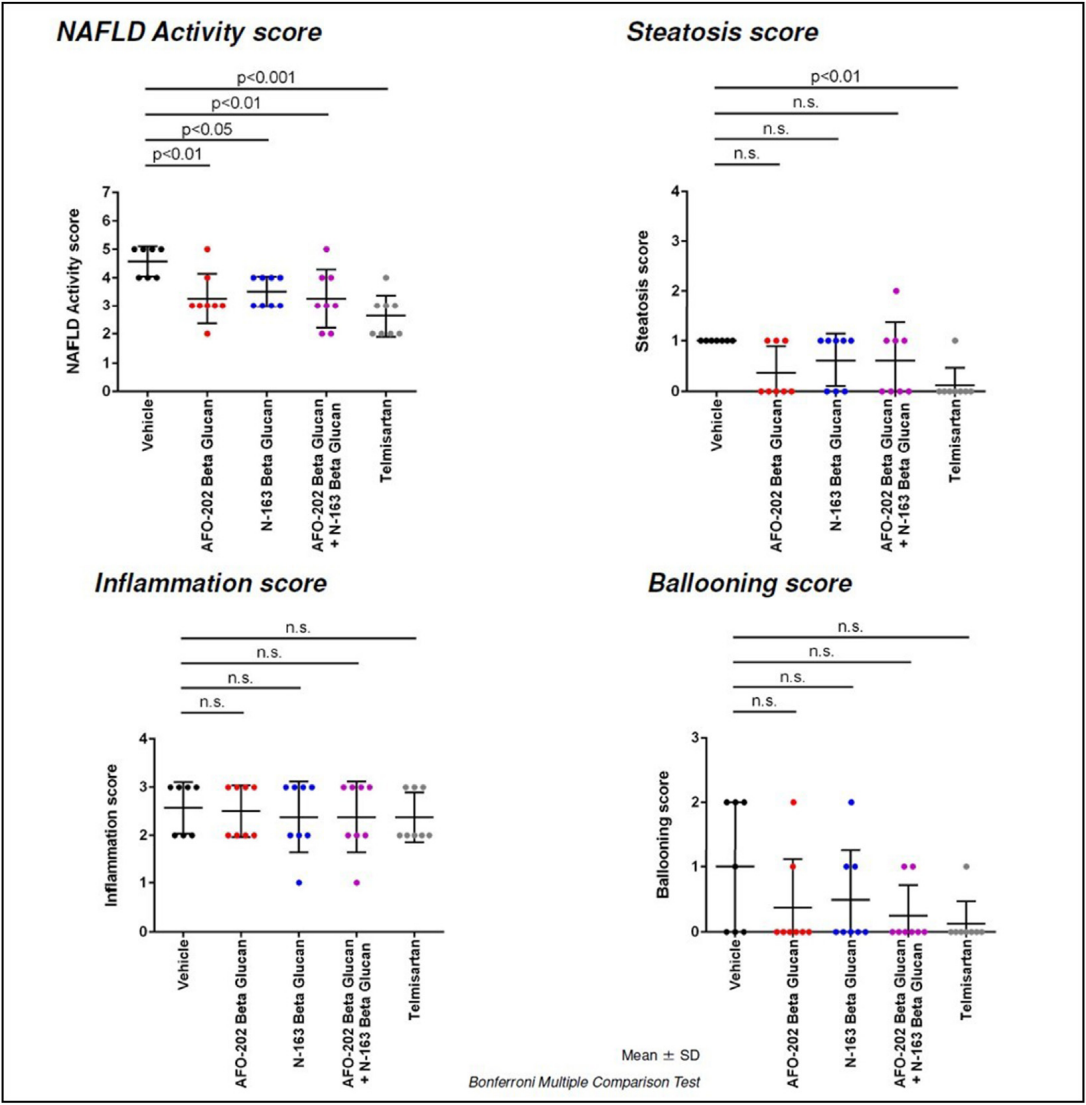
NAFLD activity score (NAS), steatosis, inflammation, and ballooning scores in the various groups based on H and E staining.

The telmisartan, N-163, and AFO-202 +N-163 groups of mice had a significantly lower NAS compared with untreated and vehicle-treated groups of mice (Mean score, telmisartan: 2.6 ± 0.7; AFO-202+N-163: 3.3 ± 1.0; N-163: 3.5 ± 0.5; AFO-202: 3.3 ± 0.9 and vehicle: 4.6 ± 0.5). The inflammation score was significantly decreased in the AFO-202+N-163 and N-163 groups compared with the telmisartan group (Figure 4, Figure 5). Ballooning and steatosis score was decreased most in the telmisartan group, but a decrease in ballooning and steatosis compared with vehicle treated mice was observed in the AFO-202 beta-glucan groups (Figure 4, Figure 5).

**Figure 6:**
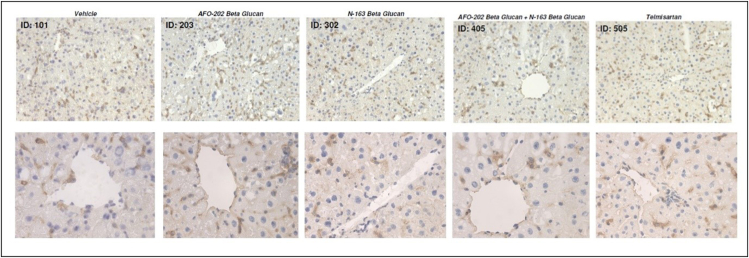
A. Representative photomicrographs of F4/80-immunostained liver sections; Upper panel: magnification: ×200; lower panel: magnification: x400.

**Figure 7:**
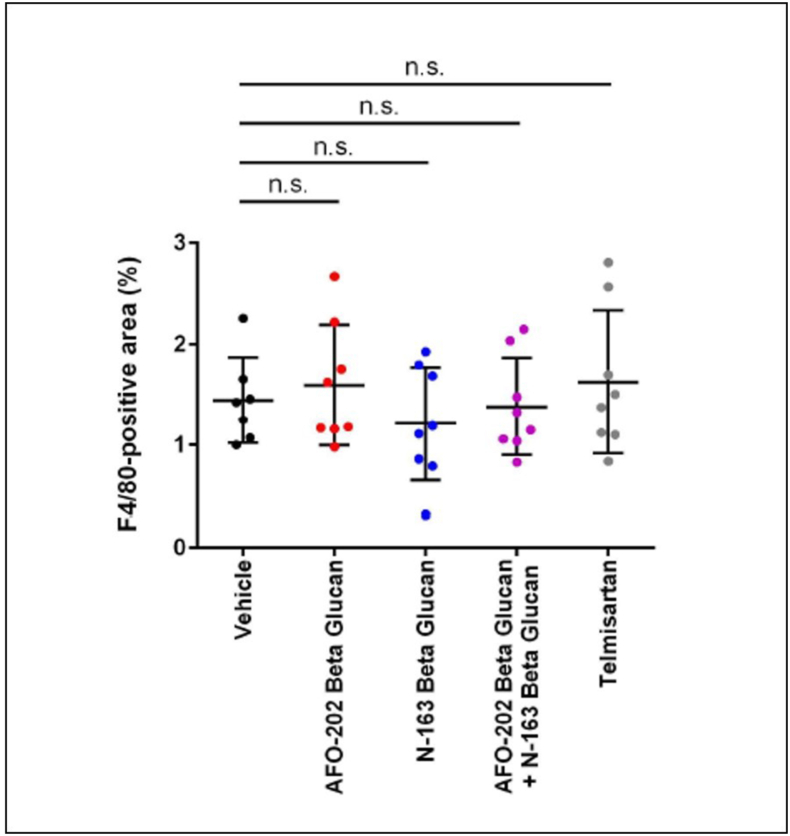
Scoring of the inflammation area by F4/80 immunostaining, which shows that the macrophage-associated inflammation was least in the N-163 group compared with the other groups.

Representative photomicrographs of F4/80-immunostained liver sections are shown in Figure 6. F4/80 immunostaining of liver sections from the vehicle group demonstrated accumulation of F4/80+ cells (macrophages associated with inflammation) in the liver lobule. F4/80 immunostaining score for these inflammatory macrophages was least in the N-163 group compared with the other groups (Figure 6, Figure 7).

## Discussion

NASH or NAFLD is a serious chronic liver disease that at first is a metabolic imbalance leading to accumulation of fat in the liver, and the inflammatory response to excess fat accumulation leads gradually to fibrosis, jeopardizing the liver function, and beyond this, if the chronic inflammation continues, it may lead to cirrhosis and hepatocellular carcinoma.^15^ Increased plasma glucose and lipid levels contribute to direct lipid deposition in the liver and lead to systemic inflammation, which contributes to the development and worsening of NAFLD.^16^ A strategic approach to NAFLD, therefore, would be first to address the metabolic imbalance, which, based on our earlier findings, could be managed by administration of AFO-202 beta-glucan,^8^, ^9^, ^10^, ^11^ while the resolution of the already established fibrosis could be addressed by N-163 beta-glucan, as shown in the present study. The present study has also proven that the combination of AFO-202 and N-163 is effective to address the chronic-inflammation-fibrosis cascade, preventing the culmination in cirrhosis or progress to carcinoma.

In this study, the effects of AFO-202 and N-163 Beta-1,3–1,6 glucans were tested individually and in combination in the STAM mice model of NASH. The decrease in body weight and liver weight was significantly reduced only in the telmisartan group (Figure 1). The inflammation and ballooning scores were decreased mainly in the AFO-202 beta-glucan groups, indicating that beta-glucans may act as an anti-inflammatory protective agent against NASH progression (Figure 4). AFO-202 beta-glucan has been shown to decrease inflammation-related cytokines in previous studies.^17^ This is further substantiated in the present study. However, fibrosis, which is the outcome of inflammation (Figure 3) and macrophage associated inflammation (F4/80 immunostaining) was reduced mainly in the N-163 group (Figure 7), and the steatosis and NAS were decreased in the AFO-202 +N-163 groups as effectively as in the telmisartan group (Figure 6). IL-6 expression which is a major proinflammatory cytokine^18^ and an independent prognostic marker for liver steatosis^19^ was markedly decreased in AFO-202 + N1-63 group (Figure 1E) indicating their application as an anti-fibrotic treatment agent in NASH.

Identifying the mechanisms behind the positive outcome observed in the study should form the major focus of further research. Beta-glucans have been reported to help in alleviating obesity by acting on modulating transcription factor peroxisome proliferator-activated receptor (PPAR)-γ.^20^ This could be one probable mechanism behind the hepatoprotective anti-inflammatory and anti-fibrotic effects of AFO-202 and N-163 in the current study. Gut microbiota, which are dysregulated in metabolic syndrome, diabetes and dyslipidemia, also lead to NASH by production of endotoxins. The prebiotic effects of the AFO-202 and N-163 beta-glucans could also contribute to NASH alleviation by helping with gut microbiota's beneficial alteration.^1^ Bacterial species, such as Proteobacteria, Enterobacteria, and *Escherichia coli* has been reported to be increased in abundance in humans with NAFLD. In a study of evaluation of gut microbiota in children with autism,^21^ AFO-202 beta-glucan has been shown to decrease the Enterobacteria and *Escherichia coli*. Evaluation of the gut microbiome and metabolome in the STAM model of NASH after beta-glucans' supplementation will throw further mechanistic insights.

AFO-202, N-163 beta-glucans and their combination are known food supplements with established safety after decades of human consumption^21^ in contrast to a pharmacological agent such as telmisartan adds to their potential for managing NAFLD. The other beneficial effects of these beta-glucans on obesity, diabetes, and dyslipidaemia^8^, ^9^, ^10^, ^11^^,^^17^^,^^22^ also provides a strong rationale for their therapeutic use in NASH related diseases.

Furthermore, the mechanisms of the minute specific details may be difficult, so additional evaluation of (i) gene expression for fibrotic and non-invasive inflammatory markers in the STAM model after AFO-202, N-163 beta-glucans administration could shed light on intricacies for a better understanding of these beta-glucans in NASH, while (ii) evaluating common markers of tissue and organ fibrosis to other organ diseases such as PPAR-γ TGFb, TNFα, MCP-1, α-SMA, TIMP-1^23^^,^^24^ could add value to examining the possibilities of an extended application of these BRMGs in lung and kidney fibrosis as well. IL-6, having been already shown to be decreased by the AFO-202+N-163 beta-glucan, which is a key cytokine implicated in inflammatory and fibrosis mechanisms^25^ of lung, liver and kidney,^26^ is a specific biomarker worth evaluation in the fibrosis of other organs in further studies.

Having been proven to be safe for human consumption as a food supplement, these two novel beta-glucans, AFO-202 studied for 25 years and N-163, a larger, multicenter study in NASH/NAFLD patients would be appropriate.

However, one has to keep in mind the limitations of this study. Although the STAM model recapitulates human NASH, an important difference is the duration taken to develop the disease and the mechanism behind. In the STAM model, the development of NASH takes place over a few weeks with the mechanism primarily being a “two-hit” hypothesis^25^^,^^26^ associated with metabolic derangements that are related to central obesity and insulin resistance. In humans, however, a multiple hit mechanism occurring over a long period of time, involving additional factors such as oxidative stress, endoplasmic reticulum (ER) stress, or presence of infectious or commensal organisms that trigger liver inflammation, pro-inflammatory cytokine (e.g. tumor necrosis factor [TNF]-α)-mediated hepatocyte injury, altered lipid partitioning and hepatotoxicity mediated by free fatty acids, abnormal intrahepatic cholesterol loading, hyperinsulinemia, hyperleptinemia, hypoadiponectinemia, apoptosis, etc.^27^^,^^28^ Furthermore, it has been reported that although this STAM model recapitulated human fatty liver disease to a great extent, there are still differences in the immunological mechanisms mediating inflammation between humans and mice, with human neutrophil-attracting chemokine IL-8 having no direct analogue in mice and differences in the corresponding immune cell subsets between mice and humans.^29^

This study was a comprehensive preclinical evaluation demonstrating the hepatoprotective anti-fibrotic effects of N-163, anti-inflammatory effects of AFO-202 beta-glucan and a combination of these two biological response modifier glucans in decreasing the NAS in an established NASH model of fatty liver disease, STAM. Considering the safety of these two food supplements, a larger clinical study in NASH patients is recommended, and further research on these beta-glucans and their beneficial effects through gene expression and common biomarkers of tissue and organ fibrosis is worthwhile, as the fundamental mechanisms of fibrosis in other organs such as the kidney and lungs have common mechanisms.

## Credit authorship contribution statement

**Nobunao Ikewaki:** Conceptualization.

**Gene Kurosawa, Masaru Iwasaki, Suryaprakash Vaddi and Vidyasagar Devaprasad Dedeepiya:** Reviewing and editing.

**Rajappa Senthilkumar**: Investigation and formal analysis.

**Gary A Levy and Senthilkumar Preethy**: Writing original draft.

**Samuel JK Abraham:** Conceptualization and writing original draft.

## Conflicts of interest

Author Samuel Abraham is a shareholder in GN Corporation, Japan which in turn is a shareholder in the manufacturing company of the Beta-Glucans described in the study.

Other authors do not have any conflict of interests.

## Acknowledgements

The authors would like to dedicate this paper to the memory of Mr. Takashi Onaka, who passed away on the June 1, 2022 at the age of 90 years, who played an instrumental role in successfully culturing an industrial scale up of AFO-202 and N-163 strains of *Aureobasidium pullulans* after their isolation by Prof. Noboru Fujii, producing the novel beta-glucans described in this study. They thank.

1. Mr. Yasushi Onaka, Mr. Masato Onaka and Dr. Mitsuru Nagataki of Sophy Inc., Japan.

2. Mr. Yoshio Morozumi and Ms. Yoshiko Amikura of GN Corporation, Japan.

3. Ms. Eiko Amemiya, II Department of Surgery, University of Yamanashi for her secretarial assistance

4. Loyola-ICAM College of Engineering and Technology (LICET) for their support to our research work.

## Funding

No external funding was received for the study.

## Availability of data and material

All data generated or analyzed during this study are included in this manuscript.
